# Case Report: Advanced prosthodontic rehabilitation using implant-assisted partial denture guided by the neutral zone concept after fibular reconstruction

**DOI:** 10.3389/fdmed.2026.1884410

**Published:** 2026-07-08

**Authors:** Panagiota Chatzidou, Athanasios Stratos, Athina Niakou, Athanasios Tsekos, Argirios Pisiotis, John Fanourgiakis, Savvas N. Kamalakidis

**Affiliations:** 1 Department of Prosthodontics, School of Dentistry, Faculty of Health Sciences, Aristotle University of Thessaloniki, Thessaloniki, Greece; 2 Private Dental Practitioner, Thessaloniki, Greece; 3 Department of Management Science and Technology, Hellenic Mediterranean University, Agios Nikolaos, Crete, Greece; 4 Department of Prosthodontics, School of Dentistry, Faculty of Health Sciences, Aristotle University of Thessaloniki, Thessaloniki, Greece; 5 Department of Prosthodontics, Tufts University School of Dental Medicine, Boston, MA, United States

**Keywords:** CBCT-guided implant planning, digital–conventional integration, fibular free-flap reconstruction, implant-assisted removable partial denture, neutral zone concept, prosthetically driven rehabilitation, reiss box attachments, stud attachments

## Abstract

Rehabilitation of partially edentulous patients following fibular free-flap reconstruction presents significant functional and biomechanical challenges due to altered soft tissue morphology, reduced vestibular depth, and compromised load-bearing capacity. In cases involving extensive tissue loss, fixed prosthetic designs may be limited in their ability to support the tongue and cheeks, while implant-assisted removable partial dentures (IARPDs) offer better hygiene access and prosthetic flexibility. This case report describes a prosthetically driven approach that integrates CBCT-guided planning with advanced conventional prosthodontic techniques. After the fibula and overlying skin graft had sufficiently healed, a customized impression protocol was used to accommodate the irregular, highly resilient grafted soft tissues. Denture tooth placement was guided by the neutral zone concept to ensure functional harmony with the surrounding muscles. A radiographic splint with barium sulfate markers enabled prosthetically guided implant planning, resulting in the placement of three implants using a fully guided approach. A removable partial denture retained by stud attachments was selected, given the extent of tissue loss and the need for improved peri-implant hygiene. Intraoral activation of the attachments stabilized the distal extension during functional loading. Because the grafted tissues were mobile, a functional impression was performed to accurately capture the prosthetic-bearing area and facilitate relining, as conventional selective pressure techniques are insufficient in such conditions. The definitive prosthesis demonstrated improved retention, occlusal harmony, and functional stability, resulting in greater patient comfort and masticatory efficiency. This case highlights the potential value of prosthetically driven planning, functional impression techniques, and neutral zone-guided tooth placement in supporting functional rehabilitation and favorable clinical outcomes in reconstructed mandibles after oncologic resection.

## Introduction

Rehabilitating patients after oncologic resection and fibular free-flap reconstruction presents considerable prosthodontic challenges. Although vascularized fibular grafts reliably restore mandibular continuity and provide a stable osseous foundation, the resulting soft-tissue morphology, altered vestibular depth, scar contracture, and reduced proprioception may compromise conventional prosthetic outcomes. Furthermore, distal extension edentulism within reconstructed segments complicates load distribution, retention, and long-term biomechanical stability (1–4).

Implant-assisted removable partial dentures (IARPDs) have emerged as a predictable treatment modality for complex clinical presentations, offering improved retention, function, and patient satisfaction compared with conventional removable prostheses (5, 6). Stud attachment systems provide favorable stress distribution (7), enhanced retention, and ease of maintenance, while facilitating oral hygiene, compared with bar-retained designs (8, 9). Clinical studies have shown that stud attachments maintain retentive performance and prosthetic stability under functional loading, contributing to long-term prosthesis success (10, 11). In reconstructed mandibles, where soft-tissue resilience and anatomical contours vary considerably, stud-retained designs may therefore be a pragmatic and patient-centered solution.

A delayed implant placement protocol is generally preferred over immediate implant insertion during fibular reconstruction for this type of oncologic lesion. However, techniques involving virtual surgical planning have also been described, in which implants are placed into the fibula prior to flap harvest, allowing them to be already integrated within the graft at the time of transfer to the mandible—a concept commonly referred to as “Jaw in a Day” (12). In oncologic surgery, the primary objective is complete resection of the disease with adequate safety margins, whereas reconstruction primarily focuses on restoring mandibular continuity rather than achieving a prosthetically ideal anatomy. As a result, the final configuration of both hard and soft tissues cannot be precisely controlled during surgery and may continue to change during healing and tissue maturation. Under these conditions, implant placement at the time of reconstruction is inherently less predictable and may compromise implant positioning and subsequent prosthetic outcomes. Delayed implant placement allows for evaluation of healed hard and soft tissues and facilitates accurate prosthetic planning, enabling optimal implant positioning, angulation, and restorative space within a prosthetically driven workflow (12, 13). Furthermore, evidence suggests that implants placed in free fibula flap–reconstructed mandibles can achieve high survival rates and functional rehabilitation when performed after adequate healing and treatment completion, supporting delayed protocols in oral oncology patients (13–15).

Contemporary rehabilitation increasingly adopts a prosthetically driven philosophy, in which definitive prosthetic requirements dictate implant position, angulation, and restorative design (16). Integrating digital diagnostics, particularly cone-beam computed tomography (CBCT) and radiographic surgical guide fabrication, enables accurate three-dimensional assessment and guided implant planning (17–19). CBCT has been shown to provide reliable linear measurements and superior anatomical detail compared with conventional imaging (20). When combined with advanced conventional prosthodontic techniques, such as impression materials with varying rigidity, border molding, altered cast impressions, and intraoral attachment pick-up procedures, a synergistic workflow can be achieved that maximizes functional and aesthetic outcomes (21).

Despite advances in digital planning, successful rehabilitation in reconstructed cases still depends on meticulous stepwise execution and careful biomechanical planning. This case report describes the prosthetically driven rehabilitation of a partially edentulous patient after fibular free-flap reconstruction, highlighting the integration of digital imaging and advanced conventional techniques to achieve a stable, functional, and individualized prosthetic outcome. This case report presents a prosthetically driven rehabilitation approach that integrates the neutral zone concept, CBCT-guided implant planning, functional impression techniques for highly mobile grafted tissues, and a stud-retained implant-assisted removable partial denture in a patient with a fibular free-flap reconstructed mandible. This combination of techniques was used to address the complex anatomical and biomechanical challenges associated with mandibular reconstruction.

## Case presentation

**Figure 1:**
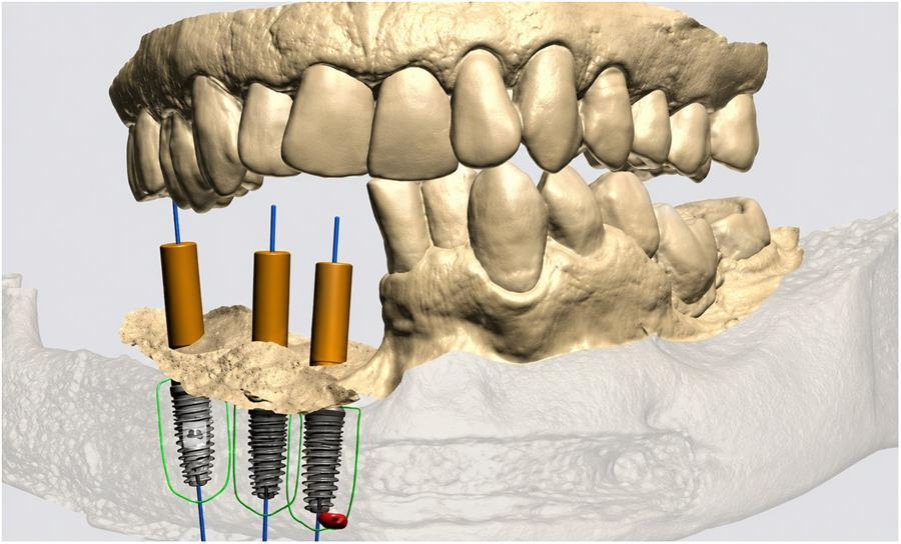
CBCT imaging and prosthetically driven digital implant planning following fibular free-flap reconstruction.

A 70-year-old patient presented 6 months after fibular free-flap reconstruction following resection of the right mandibular body for an extensive ameloblastoma, with a partially edentulous mandibular arch and missing teeth in the right mandibular segment (Figures 1).

Clinical examination revealed a natural dentition in the left mandibular segment. The maxillary arch also had a natural dentition, with no need for treatment. The occlusal relationship and available restorative space were evaluated during treatment planning. The reconstructed right mandibular segment exhibited an irregular shape, variable soft-tissue thickness, lack of keratinization, and a fibrous consistency, along with increased tissue displaceability under functional load, complicating conventional impression procedures. Additionally, key anatomical landmarks, including the retromolar pad, buccal shelf, and internal and external oblique ridges, were absent or significantly altered due to reconstruction. The patient's medical history was non-contributory, and no contraindications for implant therapy were identified. No evidence of recurrent pathology, active infection, mucosal ulceration, exposed bone, or other local conditions that could compromise implant therapy was observed during the clinical examination. Radiographic evaluation indicated sufficient bone volume for implant placement, pending three-dimensional assessment with CBCT (Figure 1). The patient's primary concerns included impaired mastication, inadequate support of the reconstructed side during function, reduced prosthesis stability, and difficulty maintaining efficient oral function. The patient expressed a desire for definitive prosthetic rehabilitation to improve comfort, function, and confidence during daily activities. The mandibular defect and associated functional limitations negatively affected the patient's social confidence and quality of life, particularly during eating and speaking in social situations.

Given the absence of reliable anatomical landmarks and the altered neuromuscular environment, treatment planning was guided by the neutral zone concept to determine the optimal denture tooth positions (22). This approach ensured that the prosthesis would be harmonized with the dynamic balance of the surrounding muscles, thereby improving prosthesis stability, patient comfort, and functional efficiency. The timeline of the prosthodontic rehabilitation and follow-up protocol is summarized in Table 1.

**Table 1:** Timeline of prosthodontic rehabilitation and follow-Up.

Stage	Procedure	Description
Initial assessment	Clinical and radiographic evaluation, 6months after mandibular reconstruction	Assessment of partially edentulous mandibular arch with natural dentition in the left mandibular segment and reconstructed fibular free-flap in the right mandibular body following ameloblastoma resection; evaluation of irregular graft morphology, altered soft tissue characteristics, and implant candidacy
	Medical history review	Confirmation of non-contributory medical history and absence of contraindications for implant therapy
Diagnostic and prosthetically driven planning phase	Preliminary impression (alginate)	Fabrication of diagnostic cast for evaluation of prosthetic space, ridge morphology, and treatment planning
	Diagnostic cast surveying and prosthesis design	Determination of path of insertion, survey lines, and undercut distribution for Kennedy Class II tooth–mucosa-supported implant-assisted removable partial denture with lingual bar and circumferential clasps
	Trial denture setup and neutral zone registration	Intraoral refinement of denture tooth arrangement using the neutral zone concept to establish optimal tooth position, polished surface contours, phonetics, esthetics, and functional stability
	Radiographic stent fabrication	Fabrication of barium sulfate–enhanced radiographic stent with occlusal pivots for prosthetically driven CBCT planning
	CBCT evaluation and implant planning	Three-dimensional radiographic assessment and prosthetically guided implant positioning based on established tooth position
Surgical phase	Guided implant placement	Placement of three implants using a fully guided surgical protocol
	Osseointegration period	Six-month healing period to allow implant integration
	Second-stage surgery, 2 months after implant placement	Placement of healing abutments for peri-implant soft tissue maturation
Definitive prosthetic phase	Rest seat preparation, 6 weeks after second-stage	Preparation of rest seats on mandibular left first and second molars to ensure support, stability, and controlled load distribution
	Preliminary impression for custom tray	Alginate impression for fabrication of individualized custom tray
	Custom tray border molding	Functional border molding with greenstick compound, with special attention to grafted tissues and altered vestibular anatomy
	Definitive anatomical impression	Polyether impression to accurately record prosthetic-bearing tissues and supporting structures
	Maxillomandibular relationship record	Fabrication of record bases and occlusal rims for jaw relation records and transfer of established neutral zone
	Master cast fabrication	Type IV stone cast fabricated from functional impression
Framework fabrication	Fabrication of cobalt–chromium implant-assisted removable partial denture framework	Framework fabrication
Denture processing	Processing with injection-molding system to minimize polymerization shrinkage and improve prosthesis adaptation	Denture processing
Attachment incorporation	Chairside pickup of stud attachments into denture base under controlled load to ensure passive fit and appropriate load transfer	Attachment incorporation
	Functional impression	Tissue conditioner applied intraorally to record grafted tissues under functional loading conditions
Prosthesis delivery	Insertion and occlusal adjustment	Verification of prosthesis fit, retention, stability, attachment function, occlusion, and patient comfort; selective flange adjustments performed as needed
Follow-up	Early follow-up (2 days)	Evaluation of soft-tissue healing, pressure areas, and initial patient adaptation
	Short-term follow-up (2 weeks)	Assessment of occlusion, attachment performance, and oral hygiene
	Intermediate follow-up (1 month)	Evaluation of prosthesis function, peri-implant tissue stability, and patient adaptation
	Long-term follow-up (6 months)	Monitoring of peri-implant health, prosthesis performance, retention, stability, patient comfort, and masticatory efficiency

### Prosthetic phase

#### Diagnostic and prosthetically driven planning phase

After adequate healing of the fibular graft and overlying skin paddle, a preliminary impression was made with alginate impression material (Alginate, Kerr) to produce a dental stone diagnostic cast (Fujirock EP, GC). Given the significant loss of hard and soft tissue volume, which limited a fixed prosthesis’ ability to provide adequate support for the tongue and cheeks and to ensure proper access for hygiene, an implant-assisted removable partial denture (IARPD) was selected as the definitive treatment.

The decision was made to fabricate a Kennedy Class II tooth–mucosa-supported implant-assisted removable partial denture, incorporating a lingual bar as the major connector and cast suprabulge circumferential clasp assemblies on the mandibular left first and second molars, engaging 0.02-inch undercuts and a rest seat at the left canine for indirect retention, serving primarily as a third reference point during the implant housing pickup procedure. The diagnostic cast was surveyed with a surveyor (JINTAI® JT-09 HOLDER) to determine the ideal path of insertion, survey line, and undercut distribution for the planned clasp assemblies.

Based on this cast, a trial denture setup was fabricated and adjusted intraorally. The neutral zone was recorded using a tissue-conditioning material applied around the trial arrangement. The patient was instructed to perform a series of functional movements, including swallowing, speaking, counting aloud, lip pursing, tongue protrusion, lateral tongue movements, and gentle sucking movements. These functional activities allowed the surrounding musculature to shape the polished surfaces and determine the optimal position of the denture teeth within the zone of muscular equilibrium. The resulting tooth arrangement (Phonares, Ivoclar Vivadent) was subsequently refined to optimize phonetics, esthetics, comfort, and prosthesis stability. This step was essential given the lack of reliable anatomical landmarks and the altered muscular balance in the reconstructed mandible.

Using this validated setup, a radiopaque stent incorporating barium sulfate markers (Bari x-ray, DS Technologies) was fabricated. The differential radiopacity enabled clear visualization of the prosthetic design on CBCT (VG One, Newtom). The stent was stabilized intraorally with occlusal pivots to ensure accurate correlation between the proposed tooth position and the underlying bone.

CBCT-guided planning enabled prosthetically driven implant placement, and three implants were placed using a fully guided surgical approach (OMNITAPER EV, 3.8 mm x 11 mm, ASTRA TECH Implant System™ EV, DENSTPLY SIRONA). A six-month unloaded healing period was selected to allow predictable osseointegration within the reconstructed fibular graft and to permit further maturation of the surrounding soft tissues before prosthetic loading. After 6 months of osseointegration, second-stage surgery was performed, and healing abutments were placed to allow soft-tissue maturation.

#### Definitive prosthetic phase

After soft tissue maturation, the definitive prosthetic phase was initiated. Based on the prior diagnostic survey, occlusal rest seat preparations were performed on the mandibular left first and second molars and a cingulum rest seat on the left canine for indirect retention before the definitive impression, to ensure appropriate retention, support, and horizontal stability for the future prosthesis.

A preliminary alginate impression (Alginate, Kerr) was obtained to fabricate a custom tray. Border molding of the custom tray was performed with greenstick compound (Green Impression Compound Sticks, Kerr) to define the prosthesis's functional extensions. Special care was taken in the grafted areas to avoid distortion, given the high resilience and mobility of the soft tissues. A definitive impression was made with polyether material (Polyether Impregum, 3M ESPE) selected for its accuracy and dimensional stability. However, conventional selective-pressure techniques are limited in reconstructed mandibles.

Record bases (Custom Impression Tray Material ALMORE) were fabricated, and the maxillomandibular relationship was recorded. The casts were mounted on a Class 3B semi-adjustable articulator (Denar Mark II, Whip Mix). The tooth arrangement was transferred and refined to align with the previously established neutral zone.

**Figure 2:**
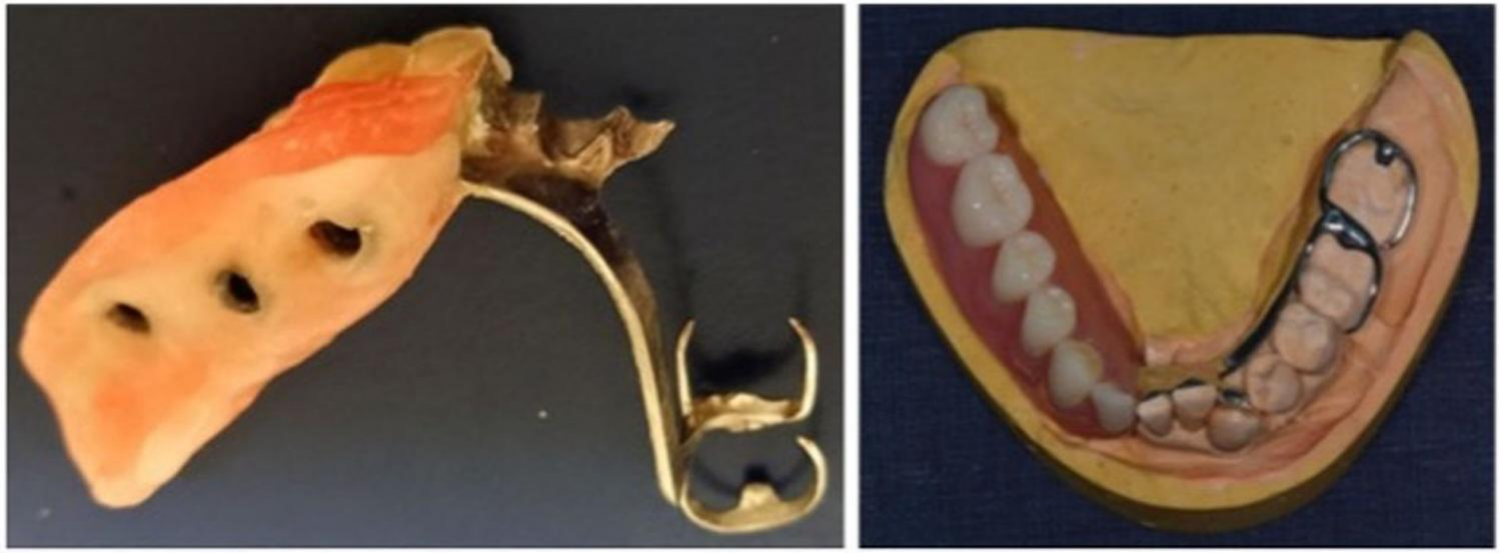
Functional impression procedure and definitive implant-assisted removable partial denture after processing and insertion.

Given the presence of compressible grafted tissues, a functional impression technique was subsequently performed to accurately record the prosthetic-bearing area (Figure 2). A viscoelastic tissue conditioner (Visco-gel, Dentsply/GC) was applied to the prosthesis's intaglio surface, and the patient was instructed to perform functional movements, including swallowing, speech, and tongue excursions. This approach recorded the tissues in their functional state and facilitated precise adaptation of the denture. The definitive master cast was fabricated using Type IV (BASE STONE, GC) stone.

A cobalt–chromium implant-assisted removable partial denture (IARPD) cobalt-chromium alloy framework (Vitalium 2000, Dentsply Sirona) was fabricated to provide rigidity and controlled load distribution across the remaining dentition and distal extension areas. The definitive prosthesis was processed with an injection-molding system (Ivobase, Ivoclar Vivadent) to minimize polymerization shrinkage and ensure an optimal fit.

**Figure 3:**
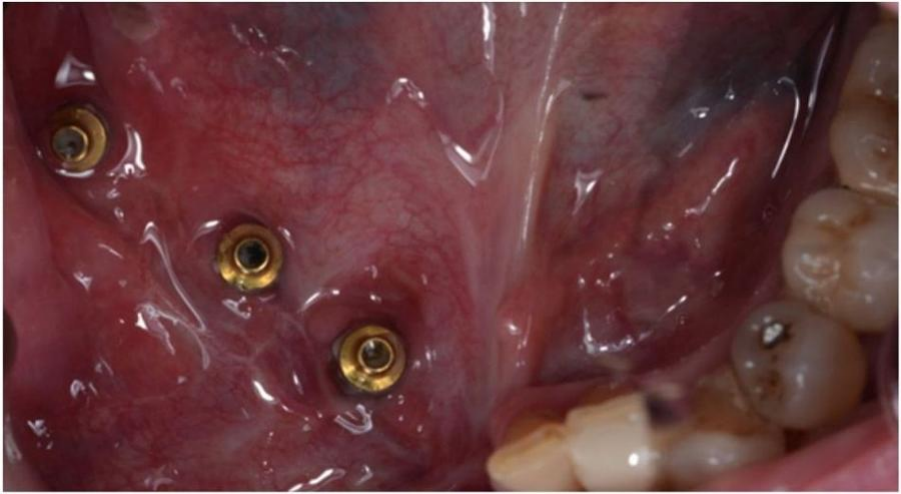
Intraoral view of reiss stud abutments after implant osseointegration and second-stage surgery.

Stud attachments (Box Stud Abutments, Reiss) were fastened to the implants with 25 N·cm of torque, as recommended by the manufacturer (Figure 3). The metal housings of the stud attachments were incorporated into the denture using a chairside pickup technique [Self-curing Acrylic Resin (Quick-Up System), VOCO] under controlled load to ensure a passive fit and appropriate load transfer. Occlusion was verified (Articulating paper, Hanel). A lingualized occlusal scheme was established to direct functional forces toward the mandibular supporting structures while minimizing lateral stresses on the reconstructed segment and supporting implants. Particular attention was given to eliminating occlusal interferences during excursive movements and achieving a stable intercuspal relationship. Post-insertion adjustments included selective relief of the lingual flange, guided by functional movements and pressure-indicating paste (Henry Schein Pressure Indicator Paste), to eliminate areas of irritation.

Follow-up evaluations demonstrated stable peri-implant tissues, improved prosthesis retention and stability, and high patient-reported satisfaction with comfort, function, and masticatory efficiency. Follow-up assessments were conducted at multiple intervals to monitor treatment outcomes. Early follow-up visits (2–7 days) focused on soft-tissue healing and initial patient adaptation. At 2 weeks, occlusion, attachment function, and oral hygiene were evaluated. The 1-month review assessed prosthesis function, peri-implant tissue stability, and patient adaptation. Long-term follow-up over 6 months monitored peri-implant health, prosthesis performance, and patient satisfaction. Overall, evaluations demonstrated stable peri-implant tissues, improved prosthesis retention and stability, and high patient-reported satisfaction with comfort, function, and masticatory efficiency throughout the observation period. No adverse biological or mechanical complications, including implant mobility, peri-implant infection, prosthesis fracture, attachment failure, or need for prosthetic component replacement, were observed during the follow-up period.

Adjunctive chemical plaque control was recommended, including 0.12% chlorhexidine mouthrinse for one week per month and daily brushing with sodium fluoride toothpaste to enhance plaque control and reduce the risk of peri-implant disease. Particular attention was directed toward evaluating the retention of the stud attachments, as functional loading and repeated insertion–removal cycles may cause wear and progressive loss of retention, necessitating timely replacement of retentive components.

## Discussion

This case underscores the pivotal role of a meticulously planned prosthetic workflow in the rehabilitation of partially edentulous patients presenting with complex anatomical and functional challenges. Optimal outcomes were predicated on prosthetically guided surgical planning, wherein osteotomies, graft positioning, and implant placement are dictated by the anticipated occlusal scheme and aesthetic objectives (23, 24). Conversely, immediate implant placement concomitant with fibular free flap reconstruction, without predefining and adhering to the tooth position, may precipitate suboptimal prosthetic emergence profiles and non-restorable or technically challenging restorative scenarios.

Cone-beam computed tomography (CBCT) has become indispensable in modern implant planning, providing three-dimensional visualization of alveolar bone, vital structures, and spatial relationships, thereby improving accuracy compared with conventional panoramic imaging (17, 20, 25). Radiographic stents containing barium sulfate in varying proportions further enhance CBCT precision, providing reliable landmarks for both surgical and prosthetic planning (1, 4).

CBCT-based planning not only enabled precise implant placement but also improved the predictability of prosthetic outcomes, thereby minimizing the risk of anatomical complications, such as floor-of-mouth hemorrhage or nerve injury (26–28). Accurate three-dimensional evaluation is particularly valuable for patients with prior surgical interventions, irregular ridge morphology, or resorption, helping to ensure long-term functional and aesthetic success (4, 29, 30). In the present case, implant placement was intentionally delayed until complete healing and stabilization of the reconstructed tissues had occurred. The CBCT evaluation demonstrated a reconstructed fibular segment with dense cortical bone and a morphology that differed substantially from the native mandible. Although the available bone volume was sufficient for implant placement, the altered ridge configuration and reconstructed soft-tissue envelope required careful prosthetically driven planning to optimize implant position, angulation, and restorative space. The dense cortical nature of vascularized fibula grafts is generally associated with favorable primary implant stability; however, differences in ridge anatomy and soft-tissue contours may complicate prosthetic design and necessitate individualized treatment planning. Delayed placement allows more accurate evaluation of the final hard- and soft-tissue architecture and facilitates prosthetically driven implant positioning. Furthermore, a six-month osseointegration period was selected because vascularized fibula grafts continue to remodel and mature following reconstruction. This approach may reduce the risk of implant malposition and support predictable prosthetic rehabilitation.

Intraoral scanning was attempted, but accurate digital acquisition was not possible because of irregular anatomical landmarks and significant soft-tissue mobility (31, 32). Consequently, reliable 3D-printed models could not be generated. Fabricating a rigid cobalt–chromium framework enabled controlled distribution of occlusal forces across teeth 31–37 and the edentulous ridge, reducing stress on the residual dentition and supporting implants (33, 34). Accurate primary impressions were obtained using a combination of red impression compound and alginate to capture both stable and mobile tissues, ensuring precise adaptation of the framework (6, 9). The functional impression technique compensated for tissue displacement under functional loading, particularly on resorbed or irregular ridges, thereby optimizing stability and retention (35, 36). These steps exemplify the integration of conventional prosthodontic techniques with advanced planning technologies, balancing mechanical stability and patient comfort.

Attachment placement using Reiss box stud abutments ensured a passive fit and predictable retention while minimizing biomechanical stress on underlying implants (6, 33, 37). Studies have shown that stud-type attachments, such as OT Equator®, maintain long-term retention and reduce prosthetic maintenance needs when correctly positioned, even in cases with implant angulation variability (38, 39). Functional loading and cyclic dislodgment have been shown to influence retention over time, highlighting the importance of meticulous prosthetic design and material selection (10, 40, 41). Several alternative treatment options were considered during treatment planning. A fixed implant-supported prosthesis was rejected because the extensive loss of hard and soft tissues would have required a large prosthetic replacement volume, potentially compromising facial support, access for hygiene, and cleansability. A bar-retained overdenture was also considered; however, the reconstructed anatomy exhibited irregular soft-tissue contours, limited vestibular depth, and variable tissue resilience. In addition, a bar design would have required greater restorative space and could have complicated oral hygiene procedures and maintenance. In contrast, stud attachments require less vertical restorative space, facilitate oral hygiene, simplify replacement of retentive components, and provide easier access for monitoring peri-implant tissues. These advantages were considered particularly relevant in the present case, where extensive soft tissue reconstruction and limited vestibular anatomy were present.

The patient initially reported discomfort after the intervention. Symptoms had significantly decreased at the two-week follow-up and had completely resolved after one month of adaptation. This improvement may be explained by neuromuscular adaptation and progressive habituation to the new prosthesis. As patients adapt to altered prosthetic contours, occlusal relationships, and sensory input, functional performance and comfort typically improve over time. Through neuroplastic mechanisms, neural circuits reorganize, and synaptic connections are modified, enabling adaptation to new conditions and reducing discomfort (42).

The long-term success of implant-assisted removable partial dentures in reconstructed mandibles depends critically on a structured maintenance and recall protocol (43–47). In this case, a 3-month recall interval was implemented to enable close monitoring of peri-implant tissues, prosthesis adaptation and occlusion, and attachment function. At each visit, oral hygiene was assessed and reinforced because non-keratinized grafted tissues are more susceptible to inflammation and plaque accumulation (43, 44, 46).

In addition, the dynamic nature of reconstructed soft tissues requires periodic assessment of denture base adaptation, with relining performed when indicated to maintain optimal support and minimize pressure-related complications (45). Regular recall also enables early detection of occlusal discrepancies and mucosal irritation, allowing prompt intervention (44, 47). Therefore, individualized maintenance protocols with short recall intervals should be considered integral to treatment planning for complex reconstructed cases, directly influencing long-term biological stability and prosthetic longevity (46, 47).

A major strength of the present report is the integration of prosthetically driven implant planning, neutral zone-guided tooth positioning, CBCT-guided surgery, and functional impression procedures for highly mobile grafted tissues. This combination enabled individualized management of the unique anatomical and biomechanical challenges associated with fibular free-flap reconstruction and contributed to the favorable functional outcome observed in this patient. The patient reported substantial improvements in comfort, prosthesis stability, masticatory function, and confidence in daily and social activities. Initial adaptation discomfort gradually resolved during follow-up, and the patient expressed high satisfaction with the functional and esthetic outcome of the rehabilitation. However, validated patient-reported outcome measures, such as the Oral Health Impact Profile (OHIP-14), General Oral Health Assessment Index (GOHAI), or visual analog scale (VAS) evaluations, were not collected. Future investigations should incorporate standardized outcome measures to objectively quantify treatment-related improvements in oral health-related quality of life and patient satisfaction. An additional limitation of this report is the relatively short follow-up period of six months. Although favorable biological and functional outcomes were observed during the observation period, longer follow-up is necessary to evaluate attachment wear, peri-implant tissue stability, prosthetic maintenance requirements, and long-term biomechanical performance in reconstructed mandibles.

Written informed consent was obtained from the patient for treatment and publication of the clinical data and accompanying images. According to institutional requirements, formal ethical committee approval was not required for publication of a single anonymized case report.

## Data Availability

The original contributions presented in the study are included in the article/Supplementary Material, further inquiries can be directed to the corresponding author.
